# Research Summary on Characterizing Impact of Environment on Adhesion of Sealed Joints in Façade Applications

**DOI:** 10.3390/ma13214847

**Published:** 2020-10-29

**Authors:** Barbora Nečasová, Pavel Liška

**Affiliations:** Faculty of Civil Engineering, Brno University of Technology, Veveří 331/95, 60200 Brno, Czech Republic; liska.p@fce.vutbr.cz

**Keywords:** artificial, adhesion, bond, cohesion, durability, frost, shear, stress, temperature, tension

## Abstract

The presented paper summarizes the main research findings on the impact of the environment concerning the durability and service life of building joint sealants. The focus is placed on sealed joints in façade applications, which can serve different purposes and can also be several meters long which often intensifies the stresses that the joint needs to withstand and therefore its service life can be significantly shortened. Different approaches, test sample geometries and high-performance sealants, were used in this context to determine the most critical aspects for the studied application sector. The research was divided into three phases where the joints were subjected to (I) artificial weathering in a laboratory environment, (II) real weathering in an external environment, and (III) weathering via a real application that was monitored for almost 4 years. The extensive research scope confirmed one commonly known presumption, that standardized artificial weathering/aging methods are not able, from a long-term perspective, to simulate the impact of a real environment. The most valuable results were obtained in the third phase of the research, where the monitoring of a real façade brought to light completely different conclusions. The joints exposed to the real environment were either completely deteriorated or showed signs of advanced aging.

## 1. Introduction

Sealing products are nowadays very often classified as “modern“ building materials. However, there are not many materials in the history of the construction industry, which would be used for completely identical or at least very similar purposes, such as sealants. Products with a similar function like modern sealants, whose historical evolution goes hand in hand with the development of adhesives, can be (according to Nicholson [1]) traced back to the stone age. A mixture of resin and mud was used to fill/seal the gaps in an effort to protect the interior against moisture intrusion and water damage [2]. The development of new products increased rapidly in the 1920s and 1940s caused by the increased demand during World War II [1,3]; and since the 1950s with the development of the chemical industry, sealing materials based on synthetic polymers became commonly available [2].

Current polymer-based sealants are one of the most common types of products used in the building sector of construction industry. They can be used for the sealing of joints and gaps in structures between individual elements/components/assemblies to provide an overall protection against the undesirable intrusion of moisture, water or air and all three at the same time [2]. Usually, they can be used as an adhesive for bonding as well. The offer of the market is extensive and selection of a product with suitable properties is very often problematic, all the more so if it would be exposed to an external environment. The outer shell of buildings creates a protective barrier against the effects of weather conditions. Therefore, it should be perfectly watertight. Although the sealing of joints in the building represents only a small percentage of the total construction, for the mentioned reasons, the selection of an appropriate material becomes a very important factor since as reported by Olsson [4], sealed joints are details that could leak the most.

**Figure 1:**
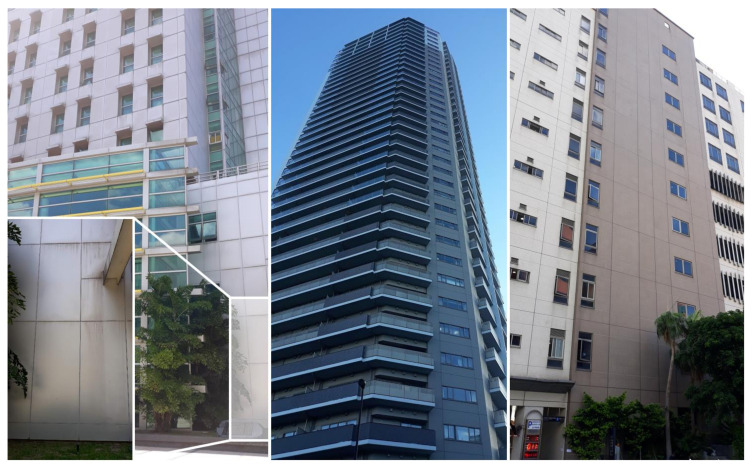
An illustrative example of sealed joints which are several meters long in façade applications.

The focus of the presented research case is placed on façade applications, where the sealed joints can be several meters long, as can be seen in Figure 1. The removal and subsequent repairs are often expensive. Thus, a long service life cycle, usually from 10 to 20 years, is expected. However, the sealant is frequently installed between two elements, i.e., façade panels, which often intensifies the stresses the joint has to endure and therefore its service life can be significantly shortened.

The presented research case was initiated by a market representative, by CIDEM Hranice, a.s., who received two complaints of a similar nature in a short period of time. In these two complaints, a problem with sealant adhesion to the cladding material was reported. The same material was used in the presented case and also the reported representatives of sealants were included in the test setup. Some of the sealants tested in the presented research case were recommended by the abovementioned client, some were chosen by the researchers. The main goal was to find the most durable solution for the tested type of cladding, since as already stated by Karpati and Sereda in 1976: “The design of joints in buildings will be successful only when the designer has adequate information to predict their movement and to choose the proper sealant.” [5].

### State of the Art

The phenomenon of the causes of sealant failure on building façades was described already by M.Y.L. Chew et al. in 1996 [6] which subsequently led to the development of an on-site diagnostic technique to test the elastic recovery of sealants [7,8] and definition of different types of causes of sealant failures [9]. The defined variants of sealant failures are now well accepted by the wider public and are commonly used for on-site failure evaluation. On the contrary, the on-site diagnostic has not been fully implemented to date. The elastic behavior and recovery of three different sealants was monitored via this in-situ technique and the short-term observation revealed that the representatives of silicones and polyurethanes show good elasticity and a high degree of movement accommodation, while polysulfide sealants are able to withstand the effect of wide temperature variations but with lower elastic recovery [6]. Moreover, the silicones had worse adherence to different substrates than other tested products.

Similar results were observed by the authors of this paper in a research project carried out in 2012–2015. Ten types of commercially available 1-K sealants (i.e., universal silicone, neutral silicone, polyurethane and MS polymer) from three different manufacturers were tested in combination with eight types of substrates (porous and non-porous). The samples were subjected to 11 standardized test methods, e.g., determination of tensile properties at maintained extension after immersion in water acc. to EN ISO 10590:2005 etc., a total of 1980 samples were tested [10]. The group of silicone sealants showed good resistance to weathering. However, the adhesion was sufficient only in combination with three types of substrates. On the contrary, polyurethanes were suitable for almost all tested materials. The most negative impact was observed in combination with MS polymer sealant [10,11,12]. One of the conclusions was that this specificity can be partly attributed to the use of a primer in combination with PU sealants that led to an improvement of adhesive properties (the tested silicones and MS polymers did not require the usage of primer). The issue of adhesion was not studied in more detail; however, a similar phenomenon was observed by Nicklisch et al. [13], again in combination with a silicone sealant. Conversely, Chew [14] stated in 2004 that perfect performance in adhesion, cohesion and elasticity was observed for PU sealant either with or without a primer, even to a concrete substrate that is considered to be one of the most challenging surfaces. Therefore, based on the previous experience and the conclusions of published works, three 1-K PU sealants were included in the presented research case.

Silicon sealants were completely excluded from the presented project. Even though structural silicones are the most traditional materials used in façade applications thanks to their good resistance to the external environment and ability to absorb joint movements around 25–30% [14,15]. A strength reduction caused by aging and temperature changes was reported by some authors [16,17,18]. Moreover, from a long-term perspective, their low elastic recovery is not satisfactory for this application area [18,19].

On the other hand, excellent resistance to artificial aging of silane terminated polymers (STP) and silyl modified polymers (MS polymers and/or SMP) was reported throughout the last decade [18,19,20,21,22,23,24]. Modified polymer sealants should be an efficient and environmentally friendly alternative to sealants based on polyurethane. Still, Bitenieks et al. [21] stated that not only can the curing period vary widely depending on the type of MS Polymer but also the kinetics of cross-linked network formation of various products can considerably affect the mechanical properties of the selected systems. The results of our project reported above support this conclusion as well. Thus, seven representatives from three different manufacturers were tested to prevent some of the mentioned problems and also to find the most suitable solution for the tested cladding material.

Finally, many authors [2,4,23], had already stated that despite the many efforts, contemporary standardized methods of accelerated aging are not able to simulate the real impact of an external environment from a long-term perspective. The prediction of the service life of sealed joints is very delicate and researchers are nowadays more often comparing the data from the laboratory and in-situ measurements. The importance of designing experiments that enable verification of various types of weathering/aging effects that correspond to field performance was confirmed by White et al. [2]. Sealants subjected to aging methods that were simulating the weathering effects separately had little effect on the joint performance while samples exposed to improved accelerated methods, that strictly simulated the real weathering, were deteriorated. Another field study provided by Olsson et al. [4] in 2018, showed that non-permeable façade solutions can be achieved only with great difficulty, moreover, depending on the façade design. In the research case, more than 90% of tested solutions failed and water leakage of the joints was monitored. This can be partly attributed to the usage of products with poor adhesion to selected substrates and partly to improper workmanship.

Although it has been noted for many years now that different laboratory techniques and methods have high potential to simulate the external environment, there remain many challenges that have not been approached yet. Based on knowledge and practical experience with the installation of sealed joints, the presented research case was thus divided into three phases where the sealed joints were subjected to (I) artificial weathering in a laboratory environment, (II) real weathering in an external environment, and (III) weathering via a real application that was monitored for almost 4 years. The main focus was placed on a comparison of the aforementioned project phases and the critical evaluation of recorded data.

## 2. Materials and Methods

### 2.1. Material Selection

The aim of the presented research case was characterizing the impact of the environment on sealed joints in façade applications. Thus, one of the most common façade systems, a rainscreen cladding system with a ventilation gap, was selected. The system is usually composed of a load-bearing structure and façade cladding. The cement bonded particleboard with smooth grey surface without any surface finish and 10 mm thick was chosen for this purpose. The material was selected based on previous experience and also due to its common availability, low price and material properties. The large-format boards were produced by pressing a mixture of wood chips (63% by volume), Portland cement (25% by volume), water (10% by volume) and hydration additives (2% by volume) [25]. This material was used in all three phases of the presented research. The pertinent information on material properties can be found in Table 1. As can be seen in Table 1, the selected material has a relatively large percentage increase in the length caused by the moisture expansion of the material, i.e., swelling. This material property is crucial for the movement capability of sealed joints and excellent for the scrutinized purpose. As clarified by Franco et al. [26], problems can arise if materials with different coefficients of thermal expansion are bonded/sealed, because of their dissimilar movement.

Two different groups of joint sealants were selected. The representatives of the first group were moisture-curing permanently elastic 1-K polyurethane sealants (hereinafter also PU sealants) with good elastic recovery properties. Products from two manufacturers were tested, see Table 2. The second tested group of joint sealants consisted of silyl/silane modified polymers (hereinafter also MS sealants). Again, only moisture-curing permanently elastic 1-K products with good elastic properties from three manufacturers were selected. More detailed information on selected products is presented in Table 3. All sealants were, according to the information provided by the manufacturer, suitable for the sealing of the selected façade cladding material and for outdoor applications. Two representatives of the 2nd group were classified as fire rated (marked with F).

### 2.2. Methods and Testing

**Figure 2:**
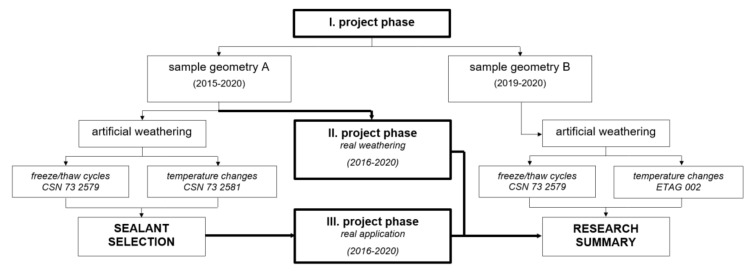
Simplified graphic illustration of the research program.

To summarize the scope of the presented research, it was divided into three phases, as can be seen in the simplified diagram in Figure 2. Depending on the research phase, different sample design and/or weathering/aging method was used.

As mentioned above, the research was initiated in 2015 by a market representative. Therefore, the first phase was conducted already in 2015 while only the sample geometry called design A was used. The details of this geometry were precisely described already in a research paper from 2017 [27]. Its novelty lies mainly in the way of the sealant application. The sealing material is installed into a gap formed between two plates/elements (i.e., façade panels) which corresponds to the real application process. Seven different joint sealants were tested, these were: PU/1/1, PU/2, STP, MS/1/1, MS/1/2, MS/1/3, MS/2/2F.

A year later, in 2016, the decision to store the test samples also in the real environment was made. This decision went hand in hand with III project phase when an opportunity to test a real façade application arose, which offered a chance of subsequent comparison of the real implementation with laboratory samples.

Later on, in 2019, based on the experience obtained from the III project phase, design B was implemented and the second part of the I project phase was conducted, see the simplified flowchart in Figure 2. This sample geometry represents a connection of the façade panels at the corners of the façade. This design can also be applied for the testing of sealed joints of a panel connection in its inner corners. Some changes of tested sealants were made and only six representatives were selected, these were PU/1/1, PU/1/2, PU/2, MS/1/3, MS/1/4F, MS/2/1. While the addition of a new sample geometry was inspired mainly by the monitoring of the real façade application, the changes in the group of the tested sealants were motivated by results obtained in the I project phase with design A. Some of the selected sealants were unsuitable for the researched area and cladding material, some were no longer produced by their manufacturers. Between the excluded products were, e.g., STP, MS/1/1 or MS/2/2F. Details will be described in the following chapters.

#### 2.2.1. Specimen Preparation

In the I and II project phase, the samples were composed of two parts with an identical dimension. These were 10-mm-thick plates 40/160 mm in size. A gap of (10 ± 2) mm was formed between these two plates. This gap was filled by the selected sealant. The thickness of the implemented sealed joint was c. (3 ± 2) mm in the center of the joint and approx. (6 ± 2) mm on the sides. To prevent the possible creation of a three-sided adhesion, a backer rod with a 12-mm diameter was inserted into the gap.

While the samples tested in the I phase were produced, matured and tested in the laboratory, the same procedures were, for the samples from the II phase, all conducted in an external environment. Thus, the samples were exposed to unpredictable production as well as curing conditions. As can be seen in Figure 2, only design A was tested in this phase of the research. Moreover, two representatives were excluded from this phase due to poor adhesive properties, these were STP and MS/2/2F.

#### 2.2.2. Artificial Weathering

In the I phase, the samples were subjected to accelerated artificial weathering after the 28-day curing period. The first used method simulated freeze–thaw cycles and is described by CSN 73 2579 [28]. The samples were subjected to 25 cycles when each cycle lasts 24 h. During one cycle the samples are stored in a cooling chamber at the temperature (−22 ± 2) °C for 18 h and afterward they are immersed into a water bath with water temperature c. (23 ± 2) °C for 6 h.

In the abovementioned I phase with design A, the test samples were subjected to a conditioning method which simulated the impact of sudden temperature changes. This method is based on the requirements of CSN 73 2581 [29]. The test samples were subjected to 15 cycles of alternate heating of samples from infrared lamps to the temperature (70 ± 2) °C and then cooled with a water shower to the temperature of (23 ± 2) °C. Even though, the samples were exposed to severe temperature shocks the effect on the durability of joint sealants was very mild. Hence, in the second phase with design B, a modification of the tension/rupture test defined by ETAG 002: Part 1 [30], in chapter 5.1.4.1.1, which also simulates the impact of temperature changes, was used. A process, similar to the freeze–thaw cycles was adopted; however, the samples were stored in the climatic chamber at the temperature (80 ± 2) °C for 18 h instead of being stored in a cooling chamber and subsequently stored in a water bath for the next 6 h. The samples were again subjected to 25 cycles of this weathering method. The data from both methods were compared to assess their effectiveness.

#### 2.2.3. Real Weathering

In the II phase, the samples were produced (author′s note: only design A) and stored in an outdoor environment for various periods. After 24 h, they were removed from a production mold and left cure statically. Some samples were removed and tested after 14 days, the second set of samples was tested after 6 months and then another one after 12 months. The last set of samples was removed in June of 2020 which was almost 4 years after their production. During this period, the changes in weather conditions were monitored and recorded.

The lowest temperature −17 °C was recorded on 7 January 2017, the highest air temperature 36 °C was monitored on 3 August 2017. Moreover, to evaluate the impact of temperature to which the façade system can be exposed, the daily temperature range as well as the minimal and maximal temperature differences were calculated. The average temperature difference was (15.64 ± 3.08) °C, the minimum difference 9 °C was recorded in January 2018 and the daily difference of 22 °C was observed in July 2019, when the temperature rose from 11 to 33 °C in only 4 h and in April 2020. The impact of the temperature change noted in April 2020 was not that severe since the temperature minimum was measured at 4:50 a.m. and the maximum was recorded at 16:32 p.m. The most critical daily differences were observed in 2019 with the average daily difference around (16.33 ± 3.60) °C. Simultaneously with the temperature, the total precipitation, wind speed and sun time were monitored. The obtained data were very similar to those recorded during the III phase.

#### 2.2.4. Real Façade Application

The last phase of the research project was the implementation of a façade system with sealed joints. The same substrate as in the I and II phase was used, i.e., 10-mm-thick particleboard. The appropriate size of the façade panels was determined on the basis of a numerical model [31]. The largest size of the panel was 915 × 1615 mm. It was expected that due to the large moisture expansion of the used material, the sealed joints would be exposed to much greater tension than that in a laboratory. The same width of the sealed joint as in the previous phases was used, i.e., 10 mm. Only one representative of sealants was selected for this application. The product was chosen on the basis of the results of the I phase.

**Figure 3:**
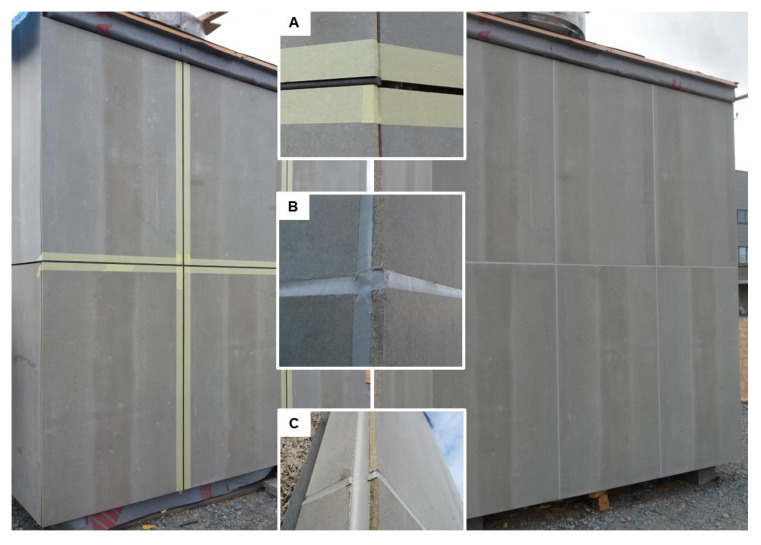
Experimental façade without (**left**) and with (**right**) sealed joints: (**A**) joint detail with backer rod, (**B**) and (**C**) detail of a connection of the horizontal and vertical joint.

As can be seen in Figure 3, the façade was oriented to three cardinal directions, i.e., the side façades to north-west and south-east, and the main front façade to the north-east, which were completely exposed to the surrounding environment. The experimental façade was built in the premises of the AdMaS research center in Brno, Czech Republic. This area is approximately 6 km far from the location where the samples were stored in II project phase. Therefore, they were exposed to similar weather conditions.

#### 2.2.5. Testing Methods

To assess the impact of the environment, all test samples from the I and II phase were subjected to a tensile test. The samples were exposed to normal stresses at a speed of c. 5.0 mm/min. The test was performed in three steps, at first, the samples were extended to 100% of their original width, if possible, they were left extended for 24 h. Subsequently, if no failure was observed, the samples were extended until their break. Only the strain behavior and mode of failure were observed for all test specimens. The focus of the presented work was placed on the analysis of the impact of the environment on adhesive properties of sealed joints. Therefore, the force needed to break the joint was not recorded. Finally, the analysis of variance (one-way ANOVA) was performed to evaluate the recorded data and to determine if they were statistically distinct. This approach should also help to analyze the impact of surface adhesive properties on the tensibility and durability of the sealed joints. Since the tensibility of the joint sealant is one of the main results of this study, the values of each reference set (RS) were compared with those obtained after the abovementioned weathering methods. The investigation of the one-factor ANOVA subsequently determined whether the difference between the mean values of tensibility is statistically significant. The value of maximum relative elongation, i.e., tensibility, was used as a response and a significance level of *p* = 0.05 was considered. Therefore, the values of the analysis smaller than 0.05 pointed out a considerable impact of the tested weathering method. Furthermore, the tensibility of the reference set that was prepared in a laboratory environment was compared with data obtained for samples that were exposed to the external environment only.

In the case of the real application, only a regular visual inspection of the sealed joint was performed.

## 3. Results and Discussion

**Figure 4:**
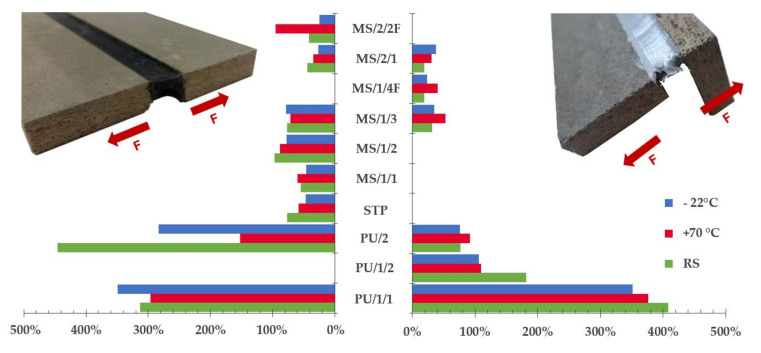
Comparison of the tensibility of test samples subjected to artificial weathering methods with the reference set: design A (**left**) and design B (**right**).

The results of the research are presented in this section. The data were evaluated separately for the three different phases. Moreover, in the I project phase two different sample geometries were tested. The mechanical behavior of sealed joints from the I and II phase of the research is described and compared. In the I and II phase, six samples were subjected to the abovementioned procedures, in the second part of the I phase where design B was tested, only five samples represented one set. The results of the putty sealant tensile tests are summarized in Figure 4.

Before the commencement of the tensile test, a normalized value was calculated for all tested representatives. The normalized value is a simple parameter that can show whether a relative shrinkage/elongation of the tested joint has appeared during its curing period and/or after conditioning. In general, the normalized value was usually around ±2% of the original joint width, no matter the type of sealant or conditioning method, i.e., laboratory or real environment. In case of PU sealants, the min and max shrinkage/elongation was around 8% which is approximately 0.92 mm. The average joint width of PU joints before the tensile test was (11.54 ± 0.35) mm. The 8% value appeared only occasionally, namely in three cases. Therefore, the impact of the environment on joint shrinkage/elongation was irrelevant. More interesting data were observed with MS sealants where even 15% shrinkage of the joint was monitored. The average joint width of MS sealants was similar to PU representatives, it was around (11.08 ± 0.61) mm. The impact of conditioning on sealed joint shrinkage was observed regularly, the most critical was the exposure of the joint to the real environment for 4 years. Representatives MS/1/1 and MS/1/3 were impacted the most. The average joint width was (9.51 ± 0.41) mm. Similar data were observed in combination with an STP sealant that was subjected to freeze–thaw cycles. Compared to the reference set, the joint width shrank by 9%.

### 3.1. Analysis of the I Project Phase

Subsequently, all test samples were subjected to a simple tensile test where the impact of the environment on the sealed joint tensibility was monitored. At first, design A was tested and eight different sealant representatives were observed, i.e., PU/1/1, PU/2, STP, MS/1/1, MS/1/2, MS/1/3, MS/2/1 and MS/2/2F.

**Figure 5:**
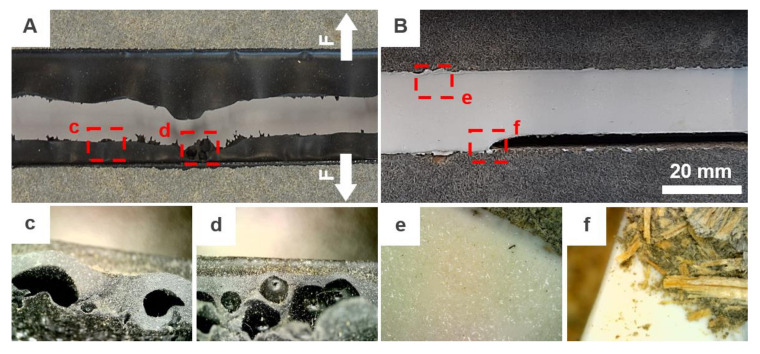
Unusual defects of (**A**) PU/2 and (**B**) MS/2/1 sealants: (**A**) premature cohesive failure of PU/2 after elongation to max. 100% with (**C**) and (**D**) typical formation of bubbles within the sealant mass; (**B**) adhesive failure of MS/2/1 with (**E**) unusual crystal-like growth effect and (**F**) partial fiber-tear.

As can be seen in Figure 4, the most critical drop of tensibility was observed in combination with PU/2 after conditioning to variable temperature changes. The tensibility was reduced by more than 65%. Unlike PU/1/1, this representative of the PU group is not bubble free product which was very often a cause of premature cohesive failure (see Figure 5a), moreover, as observed by, e.g., Banea et al. [32], the worsening of strength and elongation properties of PU adhesives/sealants caused by elevated temperature is quite common. The impact of weathering was also confirmed by the results of performed ANOVA. The *p*-value smaller than 0.05 was obtained for both conditioning methods when compared to the results of the reference set. On the contrary, this phenomenon was not observed with sample geometry design B. Nevertheless, the average elongation of the joint was almost two times smaller.

The results of another representative of PU sealants (i.e., PU/1/1) were consistent and the one-way ANOVA confirmed the irrelevance of the impact of temperature on the tensibility of the sealed joint; the p-value was higher than 0.05. Moreover, an 11.60% increase in the joint elongation was observed after freeze–thaw cycles. As stated above, the performed ANOVA has denied any negative effect of the environment on the strain behavior of the joint for both tested sample geometries. Due to these outstanding results, this representative was used in the real façade application in the III project phase.

The representative of STP sealants was tested only in the first stage of the I project phase, i.e., the design A. The material was very tough and inflexible, the joint broke even before its 100% elongation was reached, although, values close to 180% should be reached according to the manufacturer. A minor effect of the weathering procedures was monitored, even though the tensibility dropped by 23.81% after conditioning to variable temperature changes and by 38.46% after conditioning to freeze–thaw cycles. Nevertheless, according to ANOVA the impact was not significant and p-value greater than 0.05 was calculated. Comparable results were observed by Zikmundová et al. [19] and Machalická et al. [20]. Nonetheless, the structural adhesive was tested in the reported cases, thus mainly the strength reduction was evaluated. In the presented research case, the elongation and tensibility of the joint are of the main focus. Therefore, the ductility and malleability of the material are of higher importance.

Except of the STP, products of two manufacturers were represented in the group of silyl/silane-modified sealants. The MS/1/1, MS/1/3 and MS/2/1 are representatives of so-called “universal” products that can be used as sealants as well as structural adhesives. Therefore, a smaller flexibility was expected. This presumption was partially confirmed. Elongation around 100% was observed only in rare cases. On the contrary, the tensibility of MS polymers is very often much smaller than that of PU products. The behavior of MS/1/1 and MS/1/2 in the tensile test was very similar; c. 17% drop of relative elongation was observed in both cases after freeze–thaw cycles and the impact of temperature was insignificant. Moreover, these products were tested only in the first part of the project phase since their production was canceled in 2017 and they were replaced by the production of MS/1/3. The tolerance of the MS/1/3 against temperature changes was decent. The one-way ANOVA did not point out any significant deviations in the case of design A. The *p*-value larger than 0.05 was calculated. A noteworthy impact of temperature was monitored for design B when a 67% increase in joint elongation at break was recorded after the conditioning to variable temperature changes. A 12% rise of tensibility was observed also after conditioning to freeze resistance, see Figure 4.

A significant effect of temperature was monitored for design A in combination with MS/2/1 according to the performed ANOVA. The maximum recorded tensibility for the reference set was 50.3% and the average tensibility was 44.19%. Already such small relative elongation decreased by more than 23.0% after both conditioning methods. The results were not anywhere close to the elongation around 335% stated by the manufacturer. Moreover, the material was very stiff which, in the case of design B, caused the substrate failure as can be seen in Figure 5b. An unusual effect was detected on the surface of the joint sealant after conditioning to variable temperature changes. A crystal-like growth was observed as can be seen in Figure 5e. It is quite a rare phenomenon that was not studied in-depth. However, the authors assume that it might have a similar effect to crystalline waterproofing which is protecting the sealed joint against the harsh environment. Unexpected data were obtained with design B and MS/2/1, where the worst tensibility was recorded for the reference set when only 19.20% relative elongation at break was observed. This can be partly attributed to the predominant failure mode, this was a substrate failure caused by fiber-tear, see an example in Figure 5f.

**Figure 6:**
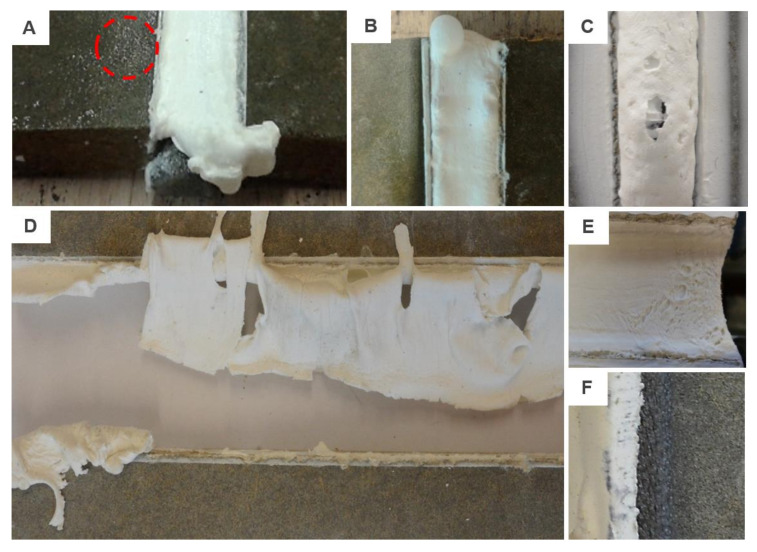
The most common defects of MS/2/2F sealant: (**A**) sample after conditioning to variable temperature changes with small particles (in the red circle) that were drained away from the sealant; (**B**) and (**C**) activation of a foaming process; (**D**) example of the typical failure of samples from the reference batch after elongation to approx. 40%; (**E**) defective surface monitored after a 14-day curing period; (**F**) backer rod glued to the sealant.

The last two products, i.e., MS/1/4F and MS/2/2F were fire-rated sealants. This was partially confirmed by the provided tests since the best results were monitored for samples exposed to high temperature (i.e., 70 and 80 °C). The tensibility of MS/1/4F increased by 116.82% and by 131.32% in case of MS/2/2F. Unfortunately, as can be seen in Figure 6, the MS/2/2F sealant is unsuitable for the studied application area. The signs of premature aging were monitored even on samples from the reference set. Furthermore, the fire protection consisted mainly of the activation of a foaming process of the putty mass which is, however, very undesirable in temperatures around +70 °C. Moreover, after contact with water, the material was completely soaked and behaved almost like a “flowing lava”. The last problem of this sealant was a formation of three-sided adhesion since it was glued to the backer rod, see Figure 6f.

### 3.2. Comparison of the I and II Project Phase

**Figure 7:**
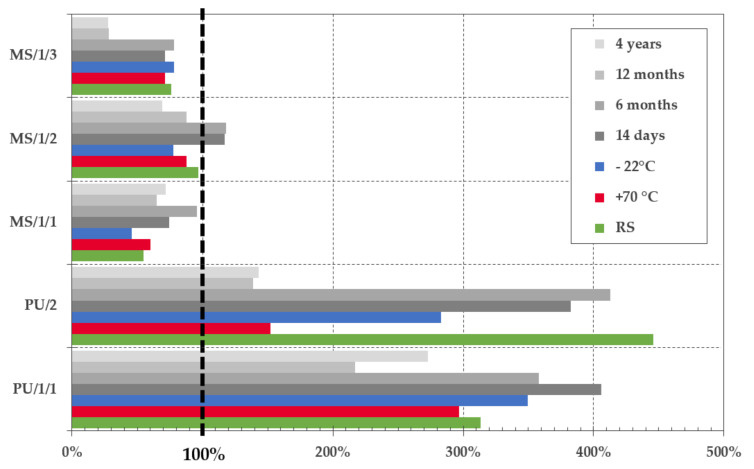
Comparison of the tensibility after artificial and real aging of samples from the I and II project phase - i.e. design A.

In the II project phase, i.e., determination of the impact of the real environment on the sealed joint durability, only five representatives from the presented group of sealants were tested. These were PU/1/1, PU/2, MS/1/1, MS/1/2 and MS/1/3. As already mentioned above, the STP and MS/2/2F were excluded from a further research due to their inappropriate properties for the studied application area. The data of tensibility presented in Figure 7, demonstrate that the additional tests performed in a real environment were very beneficial from a long-term perspective. The most crucial data were recorded in combination with the PU/2 sealant. When compared with the reference set, the one-way ANOVA showed a significant impact of the environment in all tested periods. The relative elongation of the joint dropped by more than 62%, i.e., from 445.61% (author’s note: unconditioned reference set) to 138.75% after 1-year exposure. A similar pattern was observed in combination with MS/1/3. Even though, an increase in tensibility was monitored after 14 days and 6 months, the tensile properties of samples tested after 1 and 4 years were deteriorated and dropped by more than 60% (i.e., from 76.31 to 28.05%). In the case of the sealants MS/1/1 and MS/1/2, the observed changes were not so considerable. Moreover, samples cured in an outdoor environment showed better tensile properties than those tested only in a laboratory.

Finally, the results obtained in combination with the representative PU/1/1 were the most promising. According to the performed ANOVA, the impact of the environment on the material tensibility was not significant and relative elongation larger than 200% was recorded in all combinations. The largest decrease in tensibility was observed between samples exposed to outdoor weathering for 12 months. The elongation dropped to 216.84%. The selection of this product for a real application therefore seems quite appropriate.

### 3.3. Analysis of the III Project Phase

**Figure 8:**
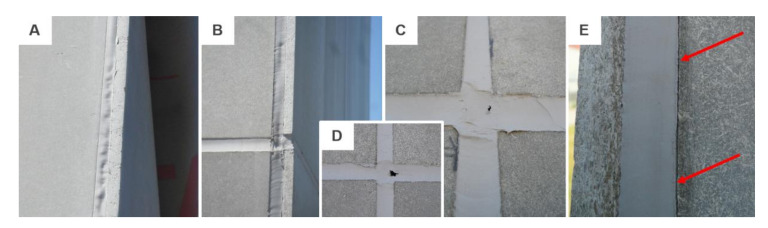
Examples of failure modes: (**A**,**B**) extrusion found on the south-east façade; (**C**,**D**) softening found on the front façade; (**E**) adhesive failure.

This project phase started in October 2016 and since then regular visual inspections were performed. Therefore, only a non-destructive analysis of the sealed joint will be described in this chapter. No significant changes were observed during the first half of the year. However, in May 2017 the regular check revealed some common modes of failure of sealants in a service. These were mainly: adhesive failure, spalling, extrusion and in two areas a problem with softening was found, see examples in Figure 8.

The extrusion of the joint was observed only in vertical joints. It was caused by the ever-changing volume of the cladding material; the sealed joint was exposed to hundreds of expansion and compression cycles. The softening of the sealant was detected only in two areas, these were located at the junction of four cladding panels. The last defect observed was an adhesive failure that was however caused by unprofessional installation. Paper tape was used during the application to protect the cladding against damage, see Figure 3 (left). Unfortunately, part of the tape was installed also in the gap, which after its removal caused an occurrence of adhesive failure. This error occurs very often in real applications.

**Figure 9:**
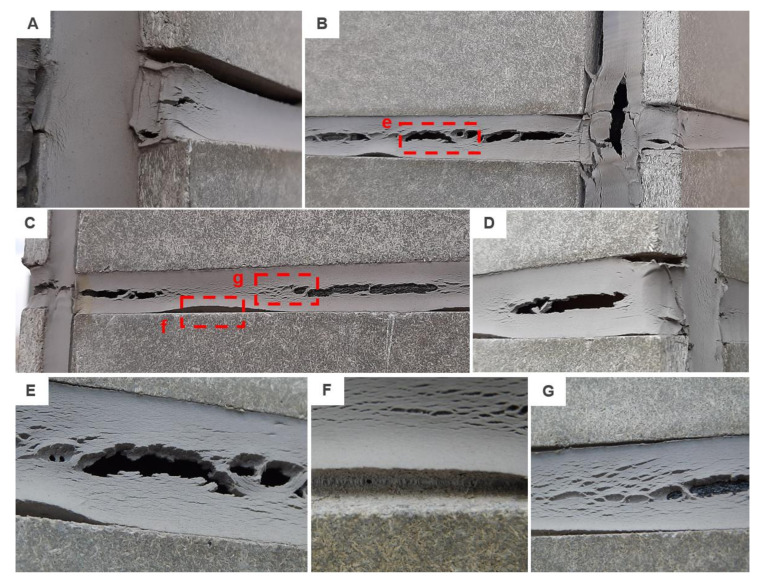
Examples of failure modes monitored after 4 years: (**A**) adhesive failure to the substrate in combination with crazing; (**B**) south-east façade and (**C**) north-west façade with cohesive failure caused by hardening as well as softening of the sealant; (**D**)–(**G**) details of observed failures.

The façade was monitored during the 4-year period. The aforesaid failures worsened but the process was by no means dramatic until the spring of 2019. The hardening and crazing of the sealed joint started to appear more often which subsequently led to extensive damage that was recorded in March 2020. A number of different failure modes were recorded. These were mainly: adhesive failure, hardening/crazing and softening in combination with cohesive failure. While all horizontal joints were completely deteriorated (see examples in Figure 9), the vertical joints were almost intact. Only the abovementioned adhesive failure was monitored. Moreover, the joints on the south-east façade were the most damaged and the least affected by the environment were the joints in the front façade that were facing the north-east. With regard to the location of the test building, it was the south-east facade that was most exposed to the weather.

### 3.4. Analysis of the Mode of Joint Failure

The scale of various common modes of failure of joint sealants in service described by Chew and Yi [6], i.e., adhesive (AF) and cohesive failure (CF) with their combination, spalling, extrusion, intrusion, hardening/crazing, softening, waisting and slumping, have been extended to include failures described by current technical standards (ČSN ISO 10365 [33]), i.e., thin-layer adhesive failure (TLF) and fiber-tear failure (FTF), which is the failure of the substrate caused by large tension in the board plane, were added.

**Figure 10:**
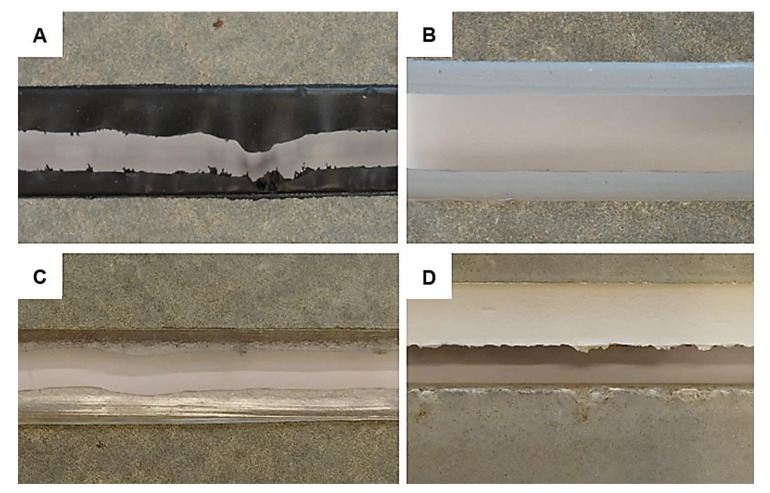
Examples of typical failure modes monitored with design A: (**A**) cohesive failure (CF) of PU/2 after more than 400% extension; (**B**) CF of PU/2/2 after extension to approximately 50%; (**C**) CF of MS/1/3 after c. 80% relative elongation; (**D**) adhesive failure (AF) of MS/1/1.

While defects such as, e.g., spalling and extrusion were observed on the real façade application, the samples from the I and II project phase were usually broken by adhesive or cohesive failure, see the examples in Figure 10. In design A, the cohesive failure was the most common mode. In combination with PU/1/1, it was recorded in 58% of tested cases, in combination with PU/2 in 89%. Both sealants manifested large flexibility and resilience to the extension. The failure of adhesion was predominant for STP (i.e., in 67% tests). A similar pattern was monitored in combination with MS sealants from the 1^st^ manufacturer: 94% with MS/1/1; 33% with MS/1/2 where CF was observed in 50% of the samples; and 83% with MS/1/3. To evaluate the failure mode of the sealant MS/2/2F was very difficult since cohesive failure of the joint was the preponderate type. However, as was demonstrated in Figure 6, the material was completely deteriorated.

**Figure 11:**
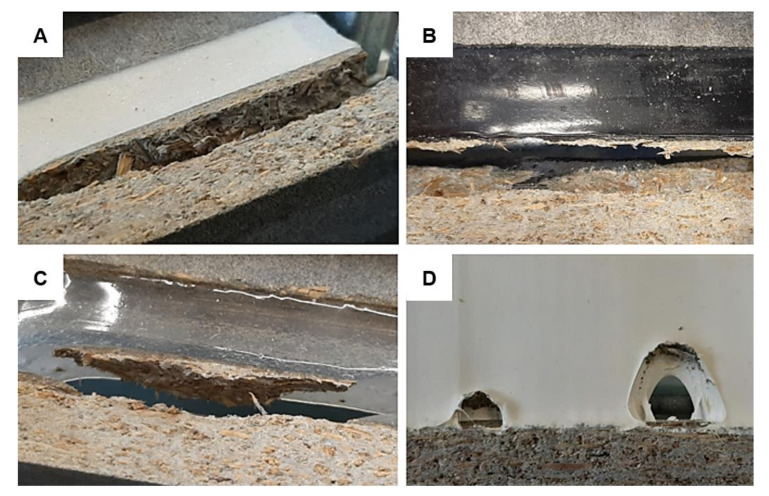
Examples of typical failure modes monitored with design B: (**A**) fiber-tear failure (FTF) of conditioned MS/2/1; (**B**) FTF of MS/1/4F; (**C**) FTF of MS/1/3 and (**D**) combination of FTF and AF of PU/1/1 after elongation to more than 350% of the original joint width.

In the case of the design B, the fiber-tear failure was a very common mode of damage, see Figure 11. It was monitored mainly in combination with MS/2/1 (see Figure 11a, it was recorded in 60% of tested cases (15 samples in total), while the sealant MS/1/3 in 13% and MS/1/4F in 7%. This type of failure was also monitored in combination with PU/2, namely in 80% of tested samples and only one specimen with PU/1/1 was broken this way; the 83% appearance of cohesive failure was recorded. In combination with PU/1/2, cohesive failure was noted in 67% of cases and adhesive failure was predominant for MS/1/4F (i.e., in 93%). Based on the presented results, it is not easy to analyze the impact of the environment of the mode of failure. However, the PU/1/1 sealant again exhibited the best properties regardless of the tested sample geometry.

## 4. Conclusions

The use of flexible polymer-based sealants for the sealing of joints and cracks has gained importance in recent years. As reported by many architects, engineers and conservationist groups [3,9], previously used sealants based on cement, gypsum or lime mortars, or vegetable oils do not meet the current requirements mainly due to their insufficient flexibility and adhesion. Flexible sealants based on synthetic polymers are able to ensure the long-lasting sealing of joints and cracks [6,14]. The sealing of joints is an essential remedy against the ingress of moisture and it is subsequently a key step to prevent the propagation of resulting cracks and the following damage to the building.

The elasticity parameter of the sealant is important for long-term tightness. Due to the effect of temperature, humidity, wind or the movement of the surrounding building components, the width of the crack or joint actually changes. These changes must be completely overcome by the sealant without losing the adhesion to the substrate or the cohesion of the used sealant. The ability of flexible sealants to overcome the changing width of joints to a certain extent while maintaining tightness is given by the value of relative elongation, i.e., tensibility.

Throughout the values of tensibility, the flexibility, resilience and perseverance of joint sealants in combination with material of large moisture expansion was described in the presented paper. The research case was initiated by a market representative who received two complaints of a similar matter in a short period of time. These complains were concerning the deterioration of sealed joint made with PU/1/1 and MS/2/2F. To better understand the failure behavior of joint sealants, two groups of sealants were studied and three phases with different conditioning methods and different sample geometry were monitored. The conclusions can be summarized as follows:

- The presented results of different types of sealants confirmed a conclusion that was published already in 1997 by Chew et al. [6], which is that there is no universal product and that different sealants are suitable for different functions, designs and environmental conditions. Even though the PU/1/1 sealant was very close to the label of the universal product, the deterioration of sealed joints in the real façade application proved exactly the opposite and confirmed the hypothesis that there is no such universal material.
- The obtained results confirmed that poor workmanship is one of the main reasons for the sealed joint failure. This fact was confirmed mainly during the third project phase, when an inappropriate installation caused a premature joint failure. The application of a layer of sealant that was too thin led to its cohesive failure and the usage of a paper tape caused the adhesive failure.
- Furthermore, the presumption that hybrid MS polymer sealants are an appropriate replacement for polyurethane products was partly disproved. The tensibility of selected MS products was much smaller than of the PU products, yet, the differences between results obtained after artificial and real weathering were smaller as well. The products might be more reliable in combination with more solid substrates.
- Moreover, the primer does not have to be used in combination with some MS sealants, e.g., in combination with STP, MS/1/3, MS/2/1 and MS/2/2F, and a very solid joint can be implemented. This is a huge advantage compared to polyurethanes since the use of primer can be extremely unsuitable in some cases, such as the application of sealants to cultural heritage structures, as they penetrate relatively deep into the substrate and are very difficult to remove.
- It was proved that the influence of the sample design, i.e., joint geometry, on the tensile stress is insignificant. Similar findings were concluded by Bues et al. [34]. Yet, premature joint failure can appear if adherends like particleboard or OSB board are connected.
- The most beneficial accomplishment of the presented project are the results of the third project phase. Although the steps recommended by the manufacturer were followed strictly, the real façade application showed much harsher failure modes than samples tested in the laboratory or samples conditioned outdoors. The results show that on-site testing methods should be included in the verification process, not only of the joint sealants as presented by Chew [6,7] or Bull et al. [23], but especially of the whole assemblies. In-situ testing and comparison with test results recorded in laboratories, as presented by, e.g., Williams et al. [35], should be discussed in the international scientific community more frequently and then integrated into new testing standards.

## Figures and Tables

**Table 1:** Basic physical and mechanical properties of the façade cladding board [25].

Material Property	Mean Values
Bulk density	1.35 g/ml
Tensile strength perpendicular to the board plane	min. 0.63 N/mm^2^
Internal bond after cycling in a humid environment	min. 0.41 N/mm^2^
Swelling thickness when stored in water for 24 h	max. 0.28%
Swelling thickness after cycling in a humid environment	max. 0.31%
Linear expansion with changes in humidity from 35 to 85% at 23 °C	max. 0.122%
Water absorption by the board when stored in water for 24 h	max. 16%
Thermal expansion coefficient	10 × 10−6 K-1
Resistance to frost at 100 cycles	R_L_ = 0.97
Mass balanced humidity at 20° and a relative humidity of 50%	9.50%

**Table 2:** Technical data and physical/mechanical properties of polyurethanes.

Material Property/ Type of Sealant ^1^	Mean Values ^2^		
	PU/1/1	PU/1/2	PU/2
Density (g/ml)	1.3	1.3	1.2
Skinning time (min)	90	60	30
Curing rate (mm/h)	2/24		3/24
Service temperature (°C)	−40 to +80	−40 to +90	−40 to +90 (+120)
Tensile strength (N/mm^2^)	1.2	1.8	1.4
Tear strength (N/mm^2^)	8.0	7.0	8.0
E-Modulus (N/mm^2^)	0.5	NA	1.0
Elongation at break (%)	600	500	400
Elastic recovery (%)	80	12.5	NA
Application conditions (°C)	+5 to +40		+5 to +35

^1^ The abbreviation PU/1/1 can be read as follows—chemical base/manufacturer/no. of the representative. ^2^ In some cases the mean values of specific material properties (e.g., curing rate) were the same for all tested products.

**Table 3:** Technical data and physical/mechanical properties of silyl/silane modified polymers.

Material Property/ Type of Sealant ^1^	Mean Values ^2^						
	STP	MS/1/1	MS/1/2	MS/1/3	MS/1/4F	MS/2/1	MS/2/2F
Density (g/l)	1.38	1.45	1.05	1.55	1.4	1.57	1.6
Skinning time (min)	20	15	10	15	10	15	5
Curing rate (mm/h)	3/24						1/168
Service temperature (°C)	−40/80	−40/100	−40/80	−40/100	−40/120	−40/90	−20/75
Tensile strength (N/mm^2^)	1.5	2.3	1.1	2.6	1.7	2.2	NA
Tear strength (N/mm^2^)	NA		2.5	NA	2.6	1.3	NA
E-Modulus (N/mm^2^)	NA				3.3	1.39	1.0
Elongation at break (%)	180	250				335	500
Elastic recovery (%)	NA	20	15		NA	25	12.5
Application conditions (°C)	+5 to +30		+5 to +35			+5 to +40	

^1^ The abbreviation MS/1/1 can be read as follows—chemical base/manufacturer/no. of the representative. ^2^ In some cases the mean values of specific material properties (e.g., curing rate) were the same for all tested products.
